# A novel nomogram for predicting the prolonged length of stay in post-anesthesia care unit after elective operation

**DOI:** 10.1186/s12871-023-02365-w

**Published:** 2023-12-07

**Authors:** Fuquan Fang, Tiantian Liu, Jun Li, Yanchang Yang, Wenxin Hang, Dandan Yan, Sujuan Ye, Pin Wu, Yuhan Hu, Zhiyong Hu

**Affiliations:** 1 Department of Anesthesiology, The First Affiliated Hospital, Zhejiang University School of Medicine, 79 Qingchun Road, Hangzhou, 310003 Zhejiang Province China; 2 Department of Anesthesiology, Ningbo Women and Children’s Hospital, Ningbo, Zhejiang China; 3 Department of Anesthesiology, Shulan Hangzhou Hospital, Hangzhou, China; 4 Cell Biology Department, Yale University, New Haven, CT USA

**Keywords:** Anesthesia, Post-anesthesia care unit, Nomogram, Enhanced recovery after surgery, Risk factors

## Abstract

**Background:**

Prolonged length of stay in post-anesthesia care unit (PLOS in PACU) is a combination of risk factors and complications that can compromise quality of care and operating room efficiency. Our study aimed to develop a nomogram to predict PLOS in PACU of patients undergoing elective surgery.

**Methods:**

Data from 24017 patients were collected. Least absolute shrinkage and selection operator (LASSO) was used to screen variables. A logistic regression model was built on variables determined by a combined method of forward selection and backward elimination. Nomogram was designed with the model. The nomogram performance was evaluated with the area under the receiver operating characteristic curve (AUC) for discrimination, calibration plot for consistency between predictions and actuality, and decision curve analysis (DCA) for clinical application value.

**Results:**

A nomogram was established based on the selected ten variables, including age, BMI < 21 kg/m^2^, American society of Anesthesiologists Physical Status (ASA), surgery type, chill, delirium, pain, naloxone, operation duration and blood transfusion. The C-index value was 0.773 [95% confidence interval (CI) = 0.765 - 0.781] in the development set and 0.757 (95% CI = 0.744-0.770) in the validation set. The AUC was > 0.75 for the prediction of PLOS in PACU. The calibration curves revealed high consistencies between the predicted and actual probability. The DCA showed that if the threshold probability is over 10% , using the models to predict PLOS in PACU and implement intervention adds more benefit.

**Conclusions:**

This study presented a nomogram to facilitate individualized prediction of PLOS in PACU for patients undergoing elective surgery.

## Introduction

A post-anesthesia care unit (PACU) is a vital part of hospital surgical suites and ambulatory care centers. The responsibility of medical staff in PACU is to transfer patients who have undergone anesthesia to the ward after satisfactory recovery [1, 2]. Prolonged length of stay in PACU (PLOS in PACU) is a combination of multiple risk factors and complications. By targeting PLOS, identifying these risk factors and complications can improve patient safety and quality of anesthesia which is beneficial to implement the protocols of enhanced recovery after surgery (ERAS) [3]. In addition, shortening the length of stay in PACU can reduce the waiting time of patients in the operating room, the turnover efficiency of which could be improved. Therefore, it is necessary to find relevant risk factors and establish a nomogram to predict the probability of PLOS in PACU. Combined with the nomogram, individualized prevention and intervention can be implemented based on risk factors.

Several clinical predictive models for different populations have been reported. Rodney A Gabriel et al. analyzed data from 4151 outpatients and identified risk factors for PLOS in PACU, including morbid obesity, hypertension, surgical specialty, primary anesthesia type, and scheduled case duration [4]. Similarly, Eric M Jaryszak et al. analyzed risk factors in 190 tonsillectomy patients [5]. Cao et al. included 913 patients and established a model for PLOS in PACU after cholecystectomy [6]. In addition, some investigations were performed on pediatric patients [7, 8]. However, the population of these studies was limited to outpatients or patients with a certain type of surgery, and the sample size was small. Most studies only analyzed relevant risk factors for PLOS in PACU without establishing and validating a nomogram for predicting the outcome. Cao et al. established a nomogram for PLOS in PACU on patients undergoing cholecystectomy [6]. However, there are many different types of surgery, and it is clear that a nomogram on a specific type of surgery is not suitable for widespread clinical use. In addition, most of the predictive variables screened in these studies were patient general characteristics and underlying diseases. Even with high predictive power, it is difficult for clinicians to prevent and intervene these risk factors.

In the current study, a nomogram was constructed to predict the PLOS in PACU by collecting clinical data on general characteristics, perioperative complications, surgery, and anesthesia from 24017 patients covering various types of surgery. We aim to provide anesthesiologists and nurses in PACU with a convenient predictive tool for the individualized prevention and intervention of associated risk factors.

## Materials and methods

### Study population

The research was in accordance with the Ethical Principles for Medical Research Involving Human Subjects, outlined in the Helsinki Declaration of 1975 (revised 2013). This study was conducted at The First Affiliated Hospital, Zhejiang University School of Medicine, a tertiary hospital. The protocol was approved by the clinical research ethic committee of the hospital(IIT20230068A). Informed consent was waived by the institutional review board due to the retrospective nature of this study. The trial was registered prior to patient enrollment in the Chinese Clinical Trial Registry (ChiCTR2300069897, Principal investigator: Fuquan Fang, https://www.chictr.org.cn/showproj.html?proj=192900, Date: 29/03/2023). This manuscript adheres to the applicable STROBE guidelines.

There are 38 elective operating rooms and 22 recovery beds in our hospital. The medical staff at PACU consists of 2 attending physicians and 10 nurses. Confusion Assessment Method is a widely used delirium assessment tool [9] and we adopt the method. The diagnosis mainly depends on four characteristics: ① acute onset of mental status changes or a fluctuating course, ② inattention, ③ disordered thinking, and ④ altered level of consciousness. Delirium can be diagnosed by meeting both ① and ②, and by having either ③ or ④. The Critical-Care Pain Observation Tool (CPOT) [10] was used for postoperative pain assessment. CPOT consists of four behavioral items : ① facial expression, ② body movements, ③ compliance with the ventilator (intubated patients) or vocalization (non-intubated patients), and ④ muscle tension. Each behavior item is scored on a scale of 0 to 2. The total score ≥ 4 was defined as pain. Bedside Shivering Assessment Scale is a four-point scale, such as absent, mild, moderate, or severe [11]. A diagnosis of chills can be made if the symptoms are mild, such as trembling of the muscles in the neck or chest. Aldrete Recovery Score ≥ 8 is considered to discharge from PACU [12]. Scores are made up of five components: activity, awareness, breathing, oxygen saturation and circulation. Each category is scored between 0 and 2. The higher the score, the better the result. RS is the arithmetic sum of the five categories. These data are stored electronically after the nurses' routine monitoring records.

Data were collected from 24017 patients undergoing elective surgery between June 2019 and February 2021. The data set only included patients over 18 years of age who were admitted to PACU for resuscitation after elective surgery with tracheal intubation anesthesia. Patients were randomly divided (7: 3) into development (16753) and validation (7264) sets. Exclusion criteria were patients with emergency surgery, brain surgery, heart surgery, mental disease, missing data. Demographic data and clinical characteristics including sex, age, Body Mass Index (BMI), hypertension, diabetes, American Society of Anesthesiologists physical status classification (ASA), type of surgery, operation duration, temperature at admission to PACU, chill, delirium, pain, naloxone, neostigmine, transfusions of red blood cells, plasma, platelets, whole blood, and autologous blood were collected through the electronic medical record system. Continuous variables were converted into categorical variables such as age, BMI, temperature, and operation duration. To determine the diagnostic reliability of age and operation duration, summary statistics were calculated including sensitivity, specificity, positive predictive value, negative predictive value, Cohen’s Kappa coefficient and the area under the receiver operating characteristic (ROC) curve. On this basis, the cut-off values of age and operative duration were calculated to be 55 and 110, respectively. BMI and temperature were transformed into multinomial variables. Therefore, the first and third quartiles were chosen as the classification basis, which were 21 kg/m^2^, 25 kg/m^2^ and 36.2℃, 37.2℃, respectively. The type of surgery was divided into minor surgery, moderate surgery, and major surgery. Minor surgeries include skin surface masses, varicose veins, thyroid nodules, breast nodules, skin debridement and endoscopic procedures such as hysteroscopy, ureteroscopy, etc. Moderate surgeries include gallstones, hepatorenal cysts, thyroid cancer, breast cancer, lung wedge resection, joint replacement, etc. Major surgeries include liver cancer, gallbladder cancer, stomach cancer, intestinal cancer, pancreatic cancer, kidney cancer, lobectomy, segmental resection, spinal surgery, etc. PLOS was defined as greater than or equal to the third quartile of length of stay (≥ 80 min).

### Statistical analysis

All statistical analyses were performed using R software (version 4.2.0). Categorical variables were described as percentages (%). whereas continuous variables were reported as medians and interquartile ranges (IQRs). The least absolute shrinkage and selection operator (LASSO) regression model was used to screen for statistically significant variables with the R package “glmnet”. These variables were then put into multivariate regression analysis with a combination method of forward selection and backward elimination based on the Akaike Information Criterion. Variables, satisfying a significance level of *P* < 0.05 were used to build predictive model. Student's t test was used to compare the area under the receiver operating characteristic curve (AUC) of model 10 and model 11.

The performance of the nomogram was evaluated for both the development and validation sets using calibration and discrimination [13]. Calibration curves were used to assess the calibration ability of this predictive nomogram, while the Harrell's concordance index (C-index) and the area under the receiver operating characteristic (ROC) curve (AUC) were both used to evaluate the discrimination ability [14]. Furthermore, decision curve analysis (DCA) was utilized to evaluate the clinical applicability [15]. Statistical significance was set at 2-sided *p* < 0.05.

## Results

### Characteristics of patients

**Fig. 1:**
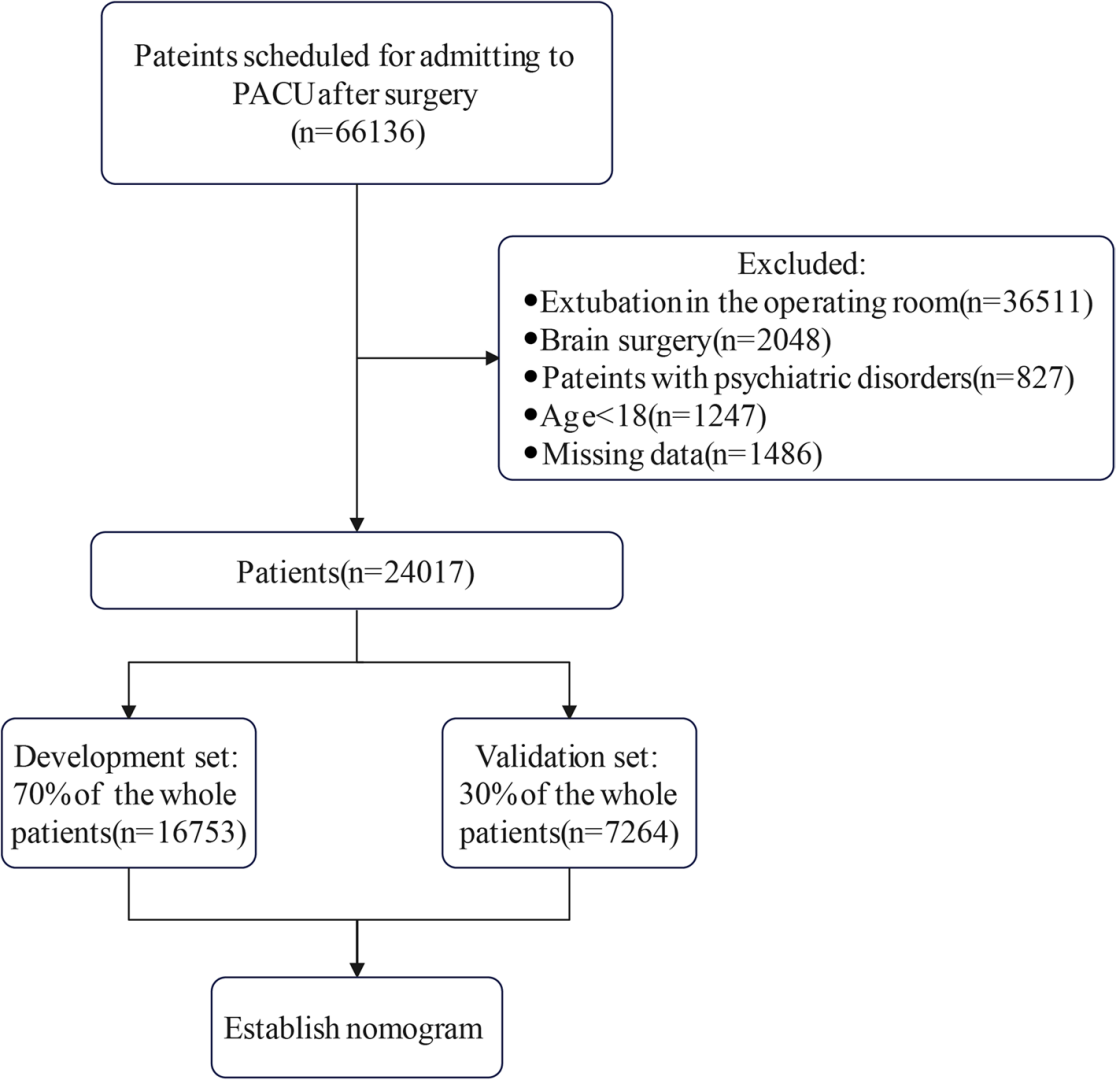
The flowchart of our study. PACU, postoperative anesthesia care unit

The data collection process was illustrated in Fig. 1. Data from the remaining 24017 cases were used for subsequent analysis. In the whole population, development, and validation sets, the median length of stay were 54 (IQR: 36-80), 54 (IQR: 36-80), and 54 (IQR: 36-80) minutes, respectively. PLOS was defined as length of stay greater than the third quartile interval. The demographic and clinical characteristics of the patients in both sets were listed in Table 1. The clinical characteristics of the total population in each variable for both the PLOS and non-PLOS groups were listed in Table 2.

**Table 1:** Clinical characteristics of the population in development and validation sets

Variables	Number(percentage,%)		*P*-value
	Development set (*n=*16753)	Validation set (*n=*7264)	
PLOS/non-PLOS	4166(24.9)/ 12587(75.1)	1797(24.7)/ 5467(75.3)	0.833
Sex (M/F)	8340(49.8)/ 8413(50.2)	3629(50.0)/ 3635(50.0)	0.811
Year (yr) <55/≥55	7340(43.8)/ 9413(56.2)	3199(44.0)/ 4065(56.0)	0.755
BMI			0.947
<21kg/m^2^	4159(24.8)	1791(24.7)	
21-25 kg/m^2^	7936(47.4)	3456(47.6)	
>25 kg/m^2^	4658(27.8)	2017(27.8)	
ASA			0.059
I/II	3543(21.1)/ 12457(74.4)	1519(20.9)/ 5455(75.1)	
III/IV	742(4.4)/ 11(0.1)	280(3.9)/ 10(0.1)	
Surgery			0.781
Minor surgery	1732(10.3)	759(10.4)	
Moderate surgery	10831(64.7)	4662(64.2)	
Major surgery	4190(25.0)	1843(25.4)	
Chill, yes	568(3.4)	267(3.7)	0.283
Delirium, yes	1079(6.4)	477(6.6)	0.732
Pain, yes	308(1.8)	121(1.7)	0.368
Temperature			0.073
<36.2℃	4191(25.0)	1720(23.7)	
36.2-36.7℃	9341(55.8)	4145(57.1)	
>36.7℃	3221(19.2)	1399(19.3)	
Naloxone	54(0.3)	29(0.4)	0.402
Neostigmine	11032(65.9)	4871(67.1)	0.07
Hyperglycemia	2363(14.1)	1100(15.1)	0.036
Hypertension	6399(38.2)	2754(37.9)	0.686
PLOD			0.494
<110 min/≥110 min	9717(58.0)/ 7036(42.0)	4248(58.5)/ 3016(41.5)	
Blood transfusion	1089(6.5)	474(6.5)	0.955
RBC	598(3.6)	262(3.6)	
Plasma	799(4.8)	334(4.6)	
Pt	19(0.1)	8(0.1)	
Cryo	6(0.0)	2(0.0)	
Whole blood	2(0.0)	0(0.0)	
Autologous blood	275(1.6)	125(1.7)	
Patients with tracheal tube discharged from PACU	13(0.0)	5(0.0)	0.820

*Abbreviations: ASA* American Society of Anesthesiologists physical status, *BMI* body mass index, *Cryo* cryoprecipitate, *PLOS* Prolonged length of stay, *PACU* postoperative anesthesia care unit, *PLOD* Prolonged length of operation duration, *Pt* platelet, *RBC* red blood cell

*P*<0.05: statistically significant

**Table 2:** The clinical characteristics of the total population in each variable for both the PLOS and non-PLOS groups

Variables	Number(percentage,%)	
	PLOS(*n=*5963)	non-PLOS (*n=*18054)
Sex (M/F)	3216(53.9)/ 2747(46.1)	8753(48.5)/ 9301(51.5)
Year (yr) <55/≥55	7340(26.5)/ 4381(73.5)	8957(49.6)/ 9097(50.4)
BMI		
<21kg/m^2^	1672(28.0)	4278(23.7)
21-25 kg/m^2^	2818(47.3)	8574(47.5)
>25 kg/m^2^	1473(24.7)	5202(28.8)
ASA		
I/II	663(11.1)/ 4860(81.5)	4399(24.4)/ 13052(72.2)
III/IV	431(7.2)/ 9(0.2)	591(3.3)/ 12(0.07)
Surgery		
Minor surgery	200(3.4)	2291(12.7)
Moderate surgery	3086(51.8)	12408(68.7)
Major surgery	2678(44.9)	3355(18.6)
Chill, yes	568(9.5)	446(2.5)
Delirium, yes	1112(18.6)	444(2.5)
Pain, yes	237(4)	192(1.1)
Temperature		
<36.2℃	1808(30.3)	4103(22.7)
36.2-36.7℃	3197(53.6)	10289(57.0)
>36.7℃	958(16.1)	3662(20.3)
Naloxone	57(1)	26(0.1)
Neostigmine	4131(69.3)	11772(65.2)
Hyperglycemia	927(15.5)	2536(14.0)
Hypertension	2754(46.2)	6399(35.4)
PLOD		
<110 min/≥110 min	2233(37.4)/ 3730(62.6)	11732(65.0)/ 6322(35.0)
Blood transfusion	616(10.3)	947(5.2)
RBC	340(5.7)	520(2.9)
Plasma	481(8.1)	652(3.6)
Pt	11(0.2)	16(0.1)
Cryo	8(0.1)	0(0.0)
Whole blood	1(0.0)	1(0.0)
Autologous blood	112(1.9)	288(1.6)
Patients with tracheal tube discharged from PACU	18(0.3)	0(0)

*Abbreviations: ASA* American Society of Anesthesiologists physical status, *BMI* body mass index, *Cryo* cryoprecipitate, *PLOS* Prolonged length of stay, *PACU* postoperative anesthesia care unit, *PLOD* Prolonged length of operation duration, *Pt* platelet, *RBC* red blood cell

*P*<0.05: statistically significant

### Nomogram variable screening

**Fig. 2:**
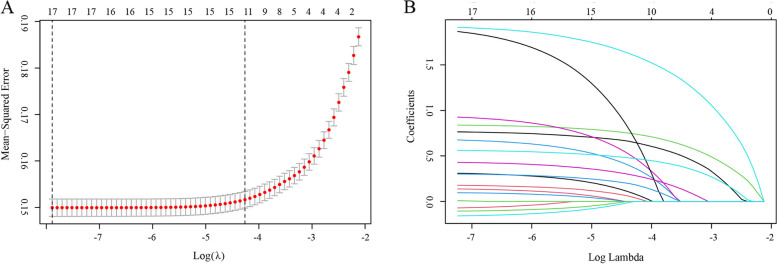
LASSO regression analysis for factors selection. **A** selection of the tuning parameter (λ) by using 10-fold via minimum and 1-SE criteria. **B** Non-zero coefficients selection by using the 10-fold cross-validation. LASSO, least absolute shrinkage, and selection operator

A total of 19 variables were entered into LASSO regression analysis after setting dummy variables for unordered categorical variables such as BMI and temperature. The LASSO result was determined with development set. The lambda. min was 0.0003744695 and 17 variables are selected, while lambda. 1se = 0.01409854, and 11 variables were selected (Fig. 2). In the current study, the lambda.1se criteria was used for predictors selection [16], and the 11 predictors included age, BMI < 21 kg/m^2^, ASA, surgery type, chill, delirium, pain, naloxone, PLOD, blood transfusion and temperature < 36.2℃. After multiple logistic regression analysis, the above factors were still statistically significant (Table 3). However, the model with 11 variables (model 11) includes hypothermia and chills. To make the nomogram simple and convenient for clinical use, we kicked out hypothermia with relatively small regression coefficient and set up a model with 10 variables (model 10). The AUC of the two models were compared and no significant difference in predictive power between the simplified model 10 and model 11 was found (*p* = 0.12). In addition, chills are defined: during recovery from anesthesia, the brain's central response to cold is reduced, and the spinal cord's central response is restored to normal, resulting in rigidity or parochial muscle tremors as a result of an involuntary nervous reflex. In this study, when chills were recorded by nurse in PACU, patients were required to present rigidity or parochial muscle tremors. The record of chills was relatively objective.

**Table 3:** Multivariable logistic regression analysis of predicting the probability of patients requiring prolonged length of stay of PACU in development set

Variables	Coef	OR(95%CI)	Z-value	*P*-value
Intercept	-5.000(-5.243--4.760)	0.007(0.005-0.009)	-40.559	<0.001
Age >55 y	0.805(0.719-0.893)	2.238(2.052-2.442)	18.141	<0.001
BMI<21kg/m^2^	0.237(0.147-0.327)	1.268( 1.158-1.387)	5.166	<0.001
ASA	0.434(0.341-0.527)	1.543(1.406-1.694)	9.149	<0.001
surgery	0.854(0.779-0.930)	2.349(2.179-2.534)	22.160	<0.001
chill	0.705(0.511-0.899)	2.024(1.666-2.457)	7.124	<0.001
delirium	1.903(1.755- 2.053)	6.707(5.786-7.790)	25.086	<0.001
pain	0.959(0.707-1.211)	2.608( 2.028-3.356)	7.466	<0.001
naloxone	1.978(1.345-2.646)	7.225(3.839-14.100)	5.993	<0.001
PLOD >110 min	0.554(0.469-0.640)	1.741(1.599-1.896)	12.736	<0.001
blood transfusion	0.327(0.181-0.473)	1.387(1.198-1.604)	4.401	<0.001
temperature<36.2 ℃	0.356(0.268-0.443)	1.428(1.308-1.558)	7.969	<0.001

Cut-point: age,<55years and ≥55years; BMI,<21kg/m^2^, 21-25kg/m^2^ and >25kg/m^2^; temperature, <36.2℃, 36.2-37.2℃ and >37.2℃; operative duration, <110min and ≥110min; The Critical-Care Pain Observation Tool score ≥ 4 was defined as pain

*Abbreviations*: *ASA* American society of Anesthesiologists Physical Status, *BMI* body mass index, *PACU* post-anesthesia care unit, *PLOD* Prolonged length of operation duration

*P*<0.05 was considered statistically significant

### Nomogram construction and validation

**Fig. 3:**
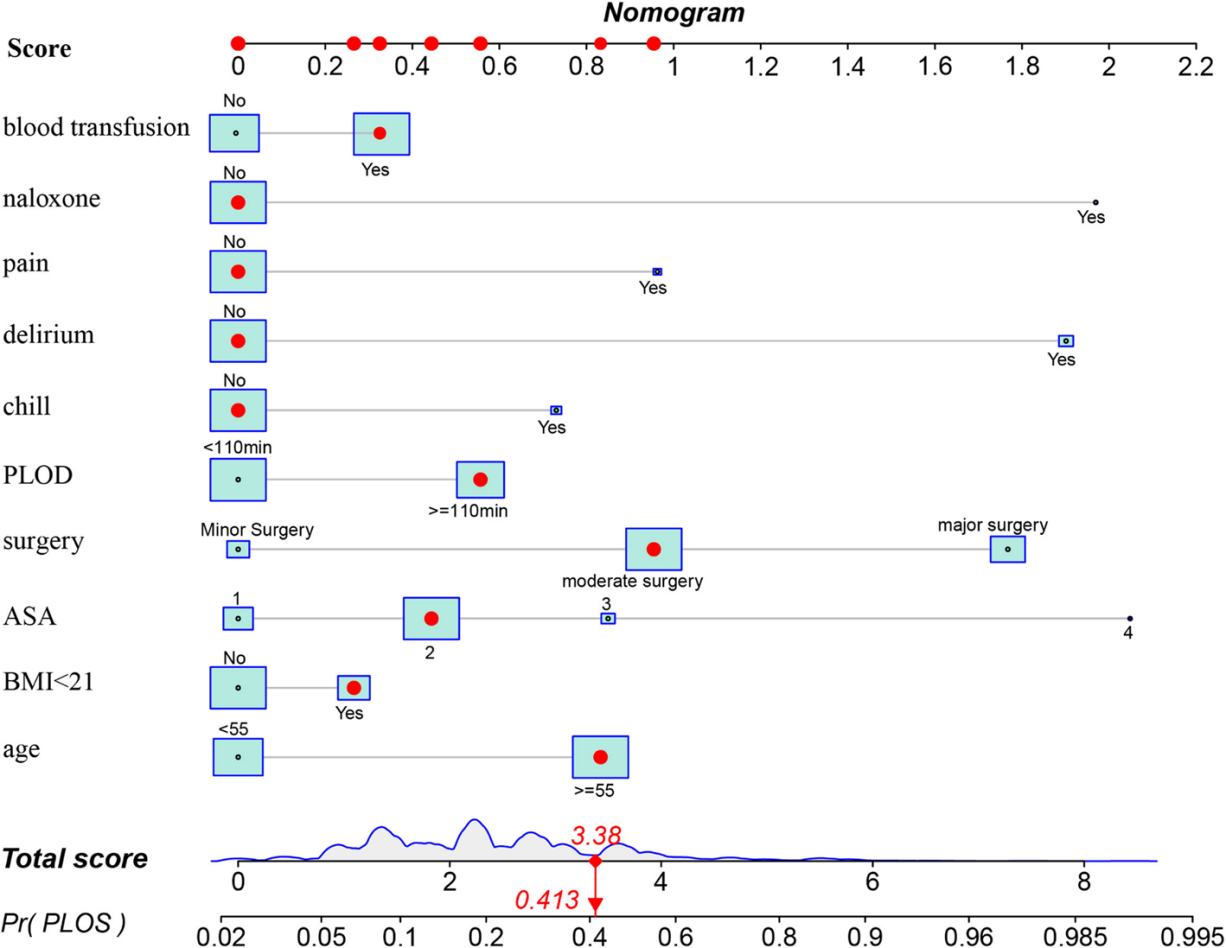
A static nomogram showing a patient's scoring process. To use the nomogram, the specific points (black dots) of individual patients are located on each variable axis. A patient's characteristics are identified by a red dot. Red dots are drawn upward to determine the score received by each variable; the sum (3.38) of these score is located on the Total score axis, and a line is drawn downward to the PLOS axes to determine the probability (41.3%) of PLOS in PACU. ASA, American society of Anesthesiologists Physical Status; BMI, body mass index; PLOD, Prolonged length of operation duration; PLOS, prolonged length of stay

**Fig. 4:**
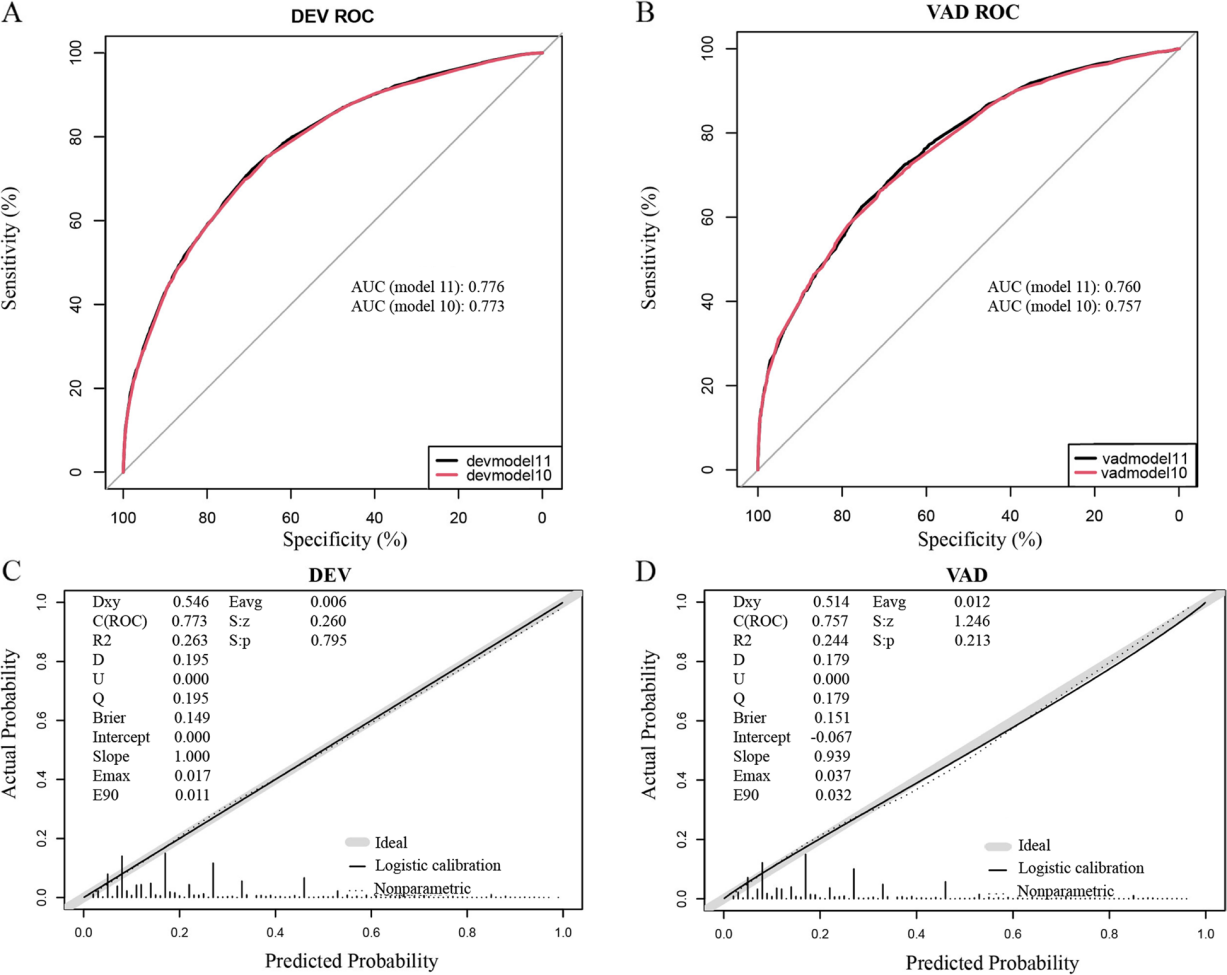
AUC and calibration curves of the models. **A**, **B** AUC of the model10 and model11 to predict probability of PLOS in PACU in the development and validation sets. The black line (model11) represents AUC = 0.776. which is considered ideal. The red line (model10, based on which the nomogram was established) represents AUC = 0.773. AUC (model10) vs AUC (model11), *p* > 0.05. **C**, **D** Calibration curves of the nomogram for PLOS in the development and validation sets. The black dots calculated with nomogram are evenly distributed around the grey bold lines, showing perfect consistency. AUC, area under the receiver operating characteristic curve; DEV, development; ROC, receiver operating characteristic curve; PLOS, prolonged length of stay; VAD, validation

A static nomogram for PLOS in PACU was constructed according to the model 10 (Fig. 3). This is an example of using the nomogram to predict the PLOS probability of a patient from data set. The total score was calculated from each item, and the predicted probability is derived accordingly. Further, a dynamic nomogram based on the model 10 was developed and freely available online to make it easier for medical staff to calculate the risk of PLOS in PACU (https://ffq11051.shinyapps.io/DynNomapp/). The regression equations constructed on Model 10 can be seen in the summary model panel of the dynamic nomogram. The C-index value of the development set was 0.773 (95% CI = 0.765-0.781), and the verification set was 0.757 (95% CI = 0.744-0.770). In both sets, the AUC was > 0.75 (Fig. 4A, B), indicating that nomogram has valuable ability to discriminate. Calibration curves of the nomogram demonstrated a high agreement between predicted and actual probabilities in both the development and validation sets (Fig. 4C, D). To sum up, the nomogram for PLOS in PACU had considerable discriminative and calibrating abilities.

### Clinical utility

**Fig. 5:**
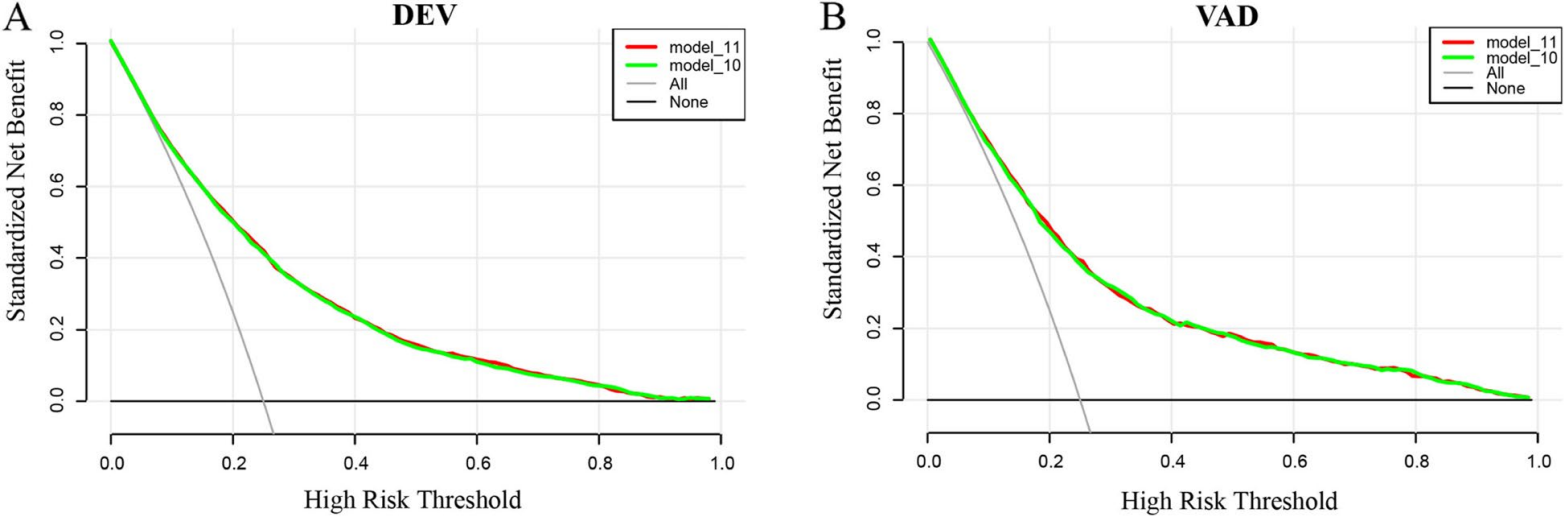
DCA of the nomogram. **A** PLOS benefit in the development set. **B** PLOS benefit in the validation set. The blue line represents model10 (based on which the nomogram was established. The red line represents model11. The results of the prediction models are the blue and red lines. The two other lines are “intervention for all” (grey line) and “intervention for none” (black line). DCA, decision curve analysis; DEV, development; PLOS, prolonged length of stay; VAD, validation

The DCA for the models was presented, which showed the comparability of model 10 and model 11 in clinical use (Fig. 5). Additionally, the DCA showed that when the threshold probability was greater than 10%, using the models to predict PLOS in PACU and implement intervention adds more benefit than either the treat-all-patients scheme or the treat-none scheme. These results further confirmed that the nomogram established based on Model 10 has a favorable performance.

## Discussion

A nomogram for predicting PLOS in PACU for patients undergoing elective surgery was developed and validated with excellent discrimination and calibration in the current study. Combined with the nomogram, individualized prevention and intervention based on the risk factors can be achieved, thereby improving operating room turnover, and implementing protocols of ERAS.

Patients with endotracheal tube into PACU were included in this study for the purpose of consistency in the start time of anesthesia recovery. Some patients may be delayed entering the PACU after extubation in the operating room due to a shortage of beds. However, these patients need only a short stay to meet the PACU discharge, which makes the stay time in PACU not an accurate reflection of recovery quality. Therefore, in order to analyze the recovery more accurately, patients with endotracheal tube were included in this study. There is no definition of "PLOS". Some studies defined it as length of stay > 2 hours [4] or > 60 minutes [17]. Rodney A Gabriel et al defined it as greater than the third quartile range [4]. Subsequent similar studies have also adopted this definition [6]. For consistency, this approach was employed in this study. Based on clinical experience or related research reports, the following risk factors statistically analyzed in this study may not be surprising, but these factors were quantified in this study to predict PLOS in PACU and were used to establish a nomogram.

Age and BMI were analyzed as risk factors. Rodney A et al. found that age ≥ 65 was not a risk factor for PLOS in PACU [4]. However, Cao et al found the opposite result in the gallbladder surgery population [6]. The reason for choosing 65 as the cutoff point was not explained in their study. In order to make the diagnosis more accurate, we determined the diagnostic reliability of an age cutoff point of 55 by calculating statistical data including sensitivity, specificity, positive predictive value, negative predictive value, Cohen’s Kappa coefficient and AUC. Age ≥ 55 was a risk factor in the study. Most studies only used dichotomies for BMI [4, 6, 18]. Therefore, low body weight had been ignored. So, we distributed body weight in quartiles, using 25% and 75% as cut-off points. This study showed that low body weight (< 21 kg/m^2^ ) was a statistically significant risk factor. Some studies had reported that BMI ≥ 40 kg/m^2^ was a risk factor for delayed postoperative recovery, mainly because morbid obesity increase the risk of sleep apnea especially in the setting of opioid utilization [4, 19]. The reason why our study did not reach a consistent conclusion may be due to the characteristics of the patient population included in this study. In this study, the number of patients with BMI ≥ 40 kg/m^2^ was 13, accounting for 0.05%, which may be caused by regional dietary and living habits. One of the five diagnostic criteria for frailty syndrome is weight loss. These patients have a poor tolerance to anesthesia and surgery, therefore requiring longer recovery time after surgery [20]. In addition, Cao et al. had reported that the lower the BMI, the longer the postoperative length of stay in PACU [6].

It is not surprising that ASA, major surgeries, and operation duration are risk factors for PLOS in PACU. Major surgeries have been thought to cause inflammatory induced postoperative cognitive decline [21], which is associated with poorer postoperative recovery, increased medical costs and higher mortality [22, 23]. Postoperative delirium increased PLOS in PACU occurrence in the study, which consisted with above. Preoperative fluid fasting for more than 6 hours was reported as an independent risk factor for delirium [24]. Incidence of cognitive decline in elderly patients under general anesthesia at bispectral index 55-65 was higher than at 40-50 [25]. Chernov and his colleagues conducted neuropsychological tests pre-operation and post-operation, which determined the association between regional cerebral blood flow reduction and poor cognitive performance [26]. For major surgery, regional nerve block combined with general anesthesia could effectively prevent cognitive decline [21], which decreased incidence of PLOS in PACU. Whether the risk of delirium differs between general anesthesia with inhalants and total intravenous anesthesia remains controversial [27]. Perioperative use of dexmedetomidine, paracetamol and Nonsteroidal Anti-inflammatory Drugs (NSAIDs) has been suggested to prevent postoperative delirium by directly alleviating neuroinflammation [28, 29]. The most relevant medications with the onset of delirium are benzodiazepines, gabapentin, and scopolamine [29]. Hypothermia is also associated with complications such as PLOS in PACU, delirium, pain, and chills [30]. Strengthening intraoperative temperature management is beneficial to improve postoperative recovery.

This study showed that pain and naloxone use were risk factors of PLOS in PACU. Anesthesiologists use different opioids depending on surgery and individual patient. By converting different opioids into morphine and then standardizing them according to the patient's weight and length of operation, this would make nomogram complicated and impractical. So, this study collected data on the use of naloxone, indirectly reflecting opioid overdose. Since both insufficient analgesia and opioid overdoses can lead to PLOS in PACU, it is necessary to explore better methods of monitoring analgesia. In recent years, various monitors have been invented for quantifying pain, such as analgesia nociception index, skin conductance, pupillometry, nociceptive flexion reflex threshold, surgical pleth index and qNOX [31]. The nociception level index, a multi-parameter artificial intelligence-driven index, can objectively guide opioid administration, potentially leading to more appropriate analgesic regimen [32]. Additionally, incorporating a multimodal analgesia approach and reducing opioid use are considered key strategy [33]. Some studies have shown that dexmedetomidine is more effective than other agents in perioperative analgesia [34–36]. However, Bhiken I Naik et al. found no such positive effect after multistage deformation-correcting spinal surgery [37]. In addition, intraoperative intravenous lidocaine (1.5 mg/kg induction followed by 2 mg/kg/h) can reduce postoperative pain and promote recovery [38]. Meta-analysis shows that NSAIDs is beneficial, such as lornoxicam, pregabalin, ibuprofen, gabapentin, and acetaminophen [39]. In a network meta-analysis of 52 trials involving 2112 subjects, the pre-emptive analgesic effect of gabapentin was evaluated as significantly reducing postoperative pain scores, opioid consumption, and the incidence of PLOS in PACU [40]. In addition to adjuvant analgesics, combined nerve block and intrathecal anesthesia are also effective [41].

This study found that intraoperative transfusion of blood products prolonged postoperative recovery. Disruption of physiological homeostasis is the main reason. In addition, transfusion of blood products had been reported to cause postoperative residual neuromuscular blockade and affect neuromuscular functional recovery in the PACU [42]. It remains unclear whether blood transfusion also affects the pharmacokinetics and pharmacodynamics of other anesthetics. Regarding blood transfusion, the "yes" and "no" were used as cut-points in the study. If a cut-point "> x units" was used as a predictor, this may improve the nomogram's predictive power. However, the variety of blood component types and quantities were collected in this study. Treating each blood component as an independent variable and counting their respective quantities would make the resulting nomogram complex and inconvenient for clinical use, especially in the environment of busy clinical anaesthetic work. In addition, this study found that neostigmine use was not a risk factor for PLOS in PACU, possibly because neostigmine does not accurately reflect neuromuscular blocker overdoses. The amount and type of neuromuscular blocker were not collected in this study, mainly to consider that if it is a risk factor, it should be standardized according to drug type, operation duration and patient weight before conducting nomogram score, which will make the nomogram complex and less useful.

The study has several limitations. First, some variables that may contribute to improving the predictive ability of the model were not included, such as cerebral apoplexy etc. Second, we did not collect detailed usage and types of opioids and neuromuscular blockers. Third, the nomogram was not verified externally. In future clinical work, we will further verify the feasibility of the model.

In summary, we have developed a predictive model with excellent differentiation and clinical utility that can help identify patients with a higher probability of PLOS in PACU. The screening of high risk factors and the establishment of nomogram are helpful to improve the quality of ERAS and the efficiency of the operating room.

## Data Availability

The data and materials in the present study can be available from the corresponding author on reasonable request. The emails of corresponding author: huzhiyong777@zju.edu.cn.

## Abbreviations

PLOS in PACU: Prolonged length of stay in post-anesthesia care unit
LASSO: Least absolute shrinkage and selection operator
AUC: Area under the receiver operating characteristic curve
DCA: Decision curve analysis
ASA: American Society of Anesthesiologists Physical Status
CI: Confidence interval
ERAS: Enhanced recovery after surgery
CPOT: Critical-care pain observation tool
BMI: Body Mass Index
IQRs: Interquartile ranges
C-index: Concordance index
NSAIDs: Nonsteroidal anti-inflammatory drugs
DEV: Development
VAD: Validation
